# The final follow-up plain radiograph is sufficient for clinical evaluation of polyethylene wear in total hip arthroplasty: A study of validity and reliability

**DOI:** 10.3109/17453674.2010.506632

**Published:** 2010-10-08

**Authors:** Maiken Stilling, Kristian Larsen, Niels T Andersen, Kjeld Søballe, Søren Kold, Ole Rahbek

**Affiliations:** ^1^Department of Orthopedics, Aarhus University Hospital; ^2^Department of Biostatistics, School of Public Health, Aarhus University, Aarhus; ^3^Orthopedic Research Unit, Department of Orthopedics, Hospital Unit West, Holstebro, Denmark

## Abstract

**Background and purpose:**

Radiostereometric analysis (RSA) is a highly accurate tool for assessment of polyethylene (PE) wear in total hip arthroplasty (THA); however, PE wear measurements in clinical studies are often limited to plain radiographs. We evaluated the agreement between PE wear measured with PolyWare software, which uses plain radiographs, and by model-based RSA, which uses stereo radiographs.

**Methods:**

Measurements of PE wear postoperatively and at final follow-up (after mean 6 years) on plain radiographs of 12 patients after cementless THA were evaluated with PolyWare software and the results were compared with those from RSA as the gold standard (Model-based RSA using elementary geometrical shape models; EGS-RSA). With PolyWare, we either used the final radiographic follow-up (PW1) only or both the postoperative follow-up and the final follow-up (PW2).

**Results:**

The 2D mean wear measured (in mm) was 0.80, 1.07, and 0.60 for the PW2, PW1, and RSA method. 2D intra-method repeatability was similar for PW1 and RSA with limits of agreement (LOAs, in mm) of ± 0.22, and ± 0.23, respectively. 2D inter-method concurrent validity was best between PW1 and EGS-RSA with LOAs of ± 0.55. For 2D linear wear measurements, the PW1 method had a clinical repeatability similar to that of RSA.

**Interpretation:**

PW1 is sufficient for retrospective determination of 2D wear from medium-term wear measurements above 0.5 mm, It alleviates the need for baseline plain radiographs, has a clinical precision similar to that of RSA, and is easy and inexpensive to use.

Wear of polyethylene (PE) components is widely regarded as the main factor limiting longevity of total hip arthroplasty (THA) (Cooper et al. 1992). Clinical studies have shown that periprosthetic osteolysis and aseptic loosening is strongly related to wear rates of above 0.2 mm/year (Sochart 1999, Dowd et al. 2000).

Radiostereometric analysis (RSA) is the most accurate tool for in vivo assessment of PE wear (Kärrholm et al. 1997, von Schewelov et al. 2004, Bragdon et al. 2006), and it is regarded as the gold standard (Ilchmann et al. 1995). However, many radiographic in vivo studies of PE wear in THA are restricted to measurements on plain radiographs because the RSA set-up is expensive and not widely available. Measurement of PE wear on plain radiographs is often limited to 2D analysis because poor quality of cross-table lateral radiographs is a common problem (Sychterz et al. 1999b, 2001). Although PE wear is known to occur multidirectionally (Yamaguchi et al. 1997, Akisue et al. 1999), the bulk of the wear is detectable on the anterior-posterior radiographs alone (Sychterz et al. 1997, Hui et al. 2003, Martell et al. 2003). Based on the availability of radiographs and investigator preferences, some authors favor analysis of serial radiographs (Sychterz et al. 1997, Kim et al. 2001, Hernigou and Bahrami 2003) to describe the pattern of wear and the steady-state wear (Sychterz et al. 1999a, Bragdon et al. 2006), whereas others use 2 radiographic follow-ups (postoperative and latest) (Kraay et al. 2006), or only the latest radiographic follow-up with the assumption of zero wear at baseline (Norton et al. 2002)

Little is known about the conformity between PE wear results measured with RSA and computerized methods using plain radiographs (Ilchmann et al. 1995, von Schewelov et al. 2004, Bragdon et al. 2006). Our group has questioned the conformity of 2D PE wear measurements based on serial, 2, or 1 radiographic follow-up (Stilling et al. 2009b). We determined that there was a statistically significant difference between all approaches, but we were unable to determine which strategy best reflected the true extent of wear (Stilling et al. 2009b). In addition, we recently showed that model-based RSA is an accurate tool for measurement of PE wear in good agreement with the true wear (Stilling 2009).

We have now studied the intra-method repeatability and concurrent validity between 2 methods (PolyWare and EGS-RSA) for measurement of PE wear in THA, in a group of patients with an average follow-up of 6 years. We wanted to determine (1) whether there would be a difference in repeatability between the methods, (2) whether there would be a difference in wear measured using 1 or 2 radiographic follow-ups with the PolyWare method, and (3) whether either of the 2 PolyWare measurement strategies (1 or 2 radiographic follow-ups) would give results similar to the wear measured by RSA (concurrent validity).

## Material and methods

The study was prepared in accordance with the Standards for Reporting of Diagnostic Accuracy (STARD) initiative (Bossuyt et al. 2003).

### Study design and patients (Table 1)

44 patients that were enrolled in an ongoing multicenter, randomized clinical trial (RCT) involving RSA had a primary THA between December 2001 and October 2005, and a subgroup of 18 patients had a minimum of 5 years of follow-up. These patients were invited for an additional clinical and radiographic double-examination follow-up linked to the present study, for measurement of 2D wear of the polyethylene liner by different methods. 12 patients with a mean follow-up of 6.1 (5.3–7.1) years responded and accepted. All investigations were conducted in accordance with ethical principles of research, informed consent was obtained from all participants, and the Central Denmark Region Committee on Biomedical Research reviewed and approved the study (Journal no. 20081096; issued December 15, 2008). Criteria for inclusion in the RCT were osteoarthritis of the hip and an age of > 18 and < 70 years. Criteria for exclusion from the RCT were osteoporosis (patients under medical treatment), neuromuscular or vascular leg disease, metabolic bone disorders, insufficient bone stock for total cementless THA, rheumatoid arthritis, malignant disease, planned pregnancy, and femoral neck fracture. 4 surgeons performed all the THAs using a posterolateral approach. Harris hip score was taken at the final follow-up.

**Table 1.** Patient demographics

Input variable	Mean	(range)
Age (years)	53	(44–65)
Height (cm)	172	(158–182)
Weight (kg)	84	(61–114)
Cup size (mm)	55	(50–62)
Polyethylene thickness (mm)	9.2	(6.8–11.8)
Follow-up (years)	6.1	(5.3–7.1)
Harris hip score at 5 years (points)	96	(84–100)
Gender ratio (males:females)	4:8	
Hip side (right:left)	7:5	

### Implants

All components (femoral stems and acetabular cups) were cementless. The femoral component was a solid Ti6A14V-alloy collarless, straight-stem Bi-Metric design (Biomet Inc, Warsaw, IN) with circumferential plasma-spray titanium and porous hydroxyapatite coating of the proximal one-quarter. The acetabular component was a plasma-sprayed titanium and hydroxyapatite-coated Mallory head, solid-finned Ringloc metal shell (Biomet). The cups were inserted using the same technique (approximately 2-mm press-fit by coating thickness, line-to-line reaming). The femoral stems were inserted by 2 alternative surgical techniques (bone rasping or bone compaction of the medullar canal) according to randomization in the RCT. The femoral heads (Biomet) were all of chrome-cobalt alloy, and they were 28 mm in diameter in 11 cases and 22 mm in diameter in one case. In all cases, the PE liners were of the Hi-Wall type and consisted of compression-molded, ultrahigh-molecular-weight PE (UHMWPE) resin, consolidated, packed, and sterilized by gamma irradiation in argon gas in the range of 2.5–4 Mrad (ArCom; Biomet).

### Radiographs

In the 2 follow-ups, all radiographs were obtained at the same hospital. The primary radiographs (stereo radiographs, anteroposterior pelvis, and cross-table lateral) were obtained during 2001 and 2003, within a week of surgery and after mobilization of the patients. The primary stereo radiographs were digital, but the plain radiographs were hard copy and were digitized to tagged image files at a resolution of 300 dots per inch at 100% scale in a high-resolution optical A3 scanner (Epson Expression 10000xl Pro A3). A standard RSA set-up of 2 synchronized ceiling-fixed roentgen tubes (Arco-Ceil/Medira; Santax Medico) angled toward each other at 40° and a uniplaner carbon calibration box (Box 24; Medis Specials, Leiden, the Netherlands) were used. At final follow-up, all radiographs were fully digital (FCR Profect CS; Fujifilm) and stored without compression. The anteroposterior and cross-table lateral radiographs had a size of 2,364 × 2,964 pixels (grayscale TIFF format) and the stereo radiographs had a size of 2,080 × 2,529 pixels (grayscale BMP format). The final radiographs were collected as double examinations by the same radiographer in January and February of 2009, with complete repositioning of the radiographic equipment and the leg of the patient between examinations (stereo radiographs, anteroposterior pelvis, and cross-table lateral). The quality of the digitized anteroposterior radiographs was generally good; however, in 3 patients the automatic circle fitting and edge detection with the PolyWare software was turned off and overruled by the manual digitizer tablet, as recommended to maintain reasonable reproducibility (Collier et al. 2003).

### Methods for PE wear measurement

**Figure 1.**
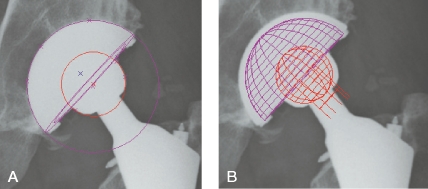
Polyethylene wear analysis with the PolyWare software. A. Circles are fitted to the peripheries of the cup and femoral head shadows B. At the end of the analysis a solid cup model is added to the output. Right hips are flipped about the vertical axis to look like left hips prior to analysis.

In the non-weight-bearing pelvic radiographs, the location of the central ray was estimated by penciling diagonals between the corners of the rectangular exposure on the radiograph. Analysis was performed by an experienced observer (LLA) with a computerized method (PolyWare Pro 3D Digital vs. 5.10; Draftware Developers, Conway, SC). This technique, developed by Devane et al. (Devane et al. 1995a, b), is only applicable to uncemented acetabular cups, and it features a digital edge-detection algorithm to fit circles and ellipses to the peripheral shadows of the femoral head and acetabular component (Figure 1). 2D PE wear is measured in the plane of the anteroposterior radiograph. At first, both the postoperative and the final radiographs were used for measurement of 2D and 3D PE wear vectors (PW2), but later only the final radiographs (PW1) were used.

**Figure 2.**
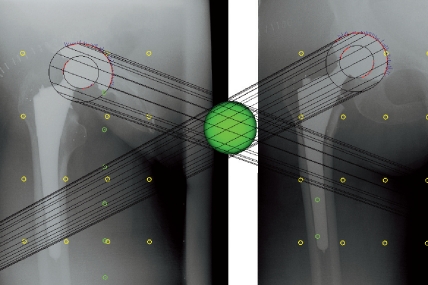
Polyethylene wear analysis in a right hip with model-based RSA, using elementary geometric shape models (EGS-RSA). Spheres are matched by an EGS algorithm to the peripheries of the femoral head and cup, and femoral head penetration is calculated as change in distance between their centers.

Both the postoperative and the final stereo radiographs were obtained without weight bearing and with the patient supine. The leg was positioned with the anatomical axis parallel to the y-axis of the calibration box. Analysis of all stereo radiographs was performed by an experienced observer (RM) with the software Model-Based RSA vs. 3.2 (Medis Specials, Leiden, the Netherlands) using elementary geometrical shape (EGS) implant models (EGS-RSA) (Kaptein et al. 2006). This is a newly developed RSA feature alleviating the need for tantalum bead marking of components or for reverse engineering of cup models (Kaptein et al. 2003). By use of the EGS mathematical algorithm in the software, software-generated sphere models were matched to the peripheries of the femoral head and cup with errors of 0.08 mm and 0.13 mm, respectively. PE wear was evaluated with the cup sphere as the reference and the femoral head sphere as the migrating (penetrating) object (Figure 2). The centers of the spheres are automatically defined by the software. The postoperative and final stereo radiographs were used for analysis. The output of EGS-RSA is a standard for RSA with 3 coordinate numbers (X, Y, and Z), and from these, 2D and 3D linear wear vectors can be calculated by Pythagoras' theorem (as the square root of (X^2^ + Y^2^) and the square root of (X^2^ + Y^2^ + Z^2^), respectively).

### Economic evaluation

A cost analysis comparing the PolyWare and RSA methods was performed with a marginal analysis (only differing costs) based on the present study; i.e., computer hardware that was necessary for both methods was not included. We defined 2 cost areas: investment costs and staff costs. The perspective of the analysis was that of the hospital. The investment costs consisted of additional X-ray equipment, calibration box, A3 transparency scanner, software, and education. X-ray equipment, calibration box, and software costs were calculated from actual costs. The staff costs consisted of the time used by the professions involved. The observed time for the radiographer to obtain 1 stereo radiograph was 30 min and 10 min for 1 AP pelvis plain radiograph. The observed time for retrieval and storage of 1 digital radiograph from the database at the radiology department was approximately 15 min, and the observed average time for finding 1 archived hard-copy radiograph and digitizing it in the transparency scanner was 45 min. RSA analysis took 90 min per patient (2 stereo radiographs) and PolyWare analysis took 30 min per patient (2 plain AP radiographs). Hourly salary for the radiographers (35 €) and for the research assistants (51 €) was obtained from the annual salary divided by 1,516 h, which was estimated by the administrative office to be the average number of effective working hours. All costs are based on 2010 prices.

### Statistics

#### Repeatability

The standard deviation of the difference (SD_dif-intra_) between the first and the second measurements (double examinations) within a method along with limits of agreement (LOA_intra_ = SD_dif-intra_ × ±1.96) were calculated according to Bland and Altman (1986). The systematic variation (bias) between the double examinations followed a normal distribution (Shapiro-Wilk test (Altman 1995)) and were tested with a paired t-test. The measures of repeatability (SD_dif-intra_ or equivalent the width of LOA_intra_) of the 3 methods were compared pairwise by looking at the ratios, and tested with an F-test. LOA_intra_ provides the same measure as the bias ± the 95% repeatability limit that is specified in the ASTM 177 standard practice for bias and precision (2008). For comparison of RSA precision with that in the literature, we calculated the 95% confidence interval (CI) for translation values of each axis.

#### Concurrent validity

Concurrent validity defines the chronological correlation between 2 measurement methods (International Epidemiological Association Inc. 1995). The RSA method was considered to be the “gold standard”. An average value from double examinations was calculated and used to estimate the bias between methods. The bias followed a normal distribution (Shapiro-Wilk test) and was tested with a paired t-test. Furthermore, the standard deviation of the difference (SD_dif-inter_) between methods and the agreement limits between methods (LOA_inter_) were calculated according to Altman (1995) (LOA_inter_ = SD_dif-inter_ × ±1.96).

Statistical significance was assumed at p < 0.05. Intercooled Stata software version 10.0 (StataCorp, College Station, TX) was used for statistical computations.

## Results

**Figure 3.**
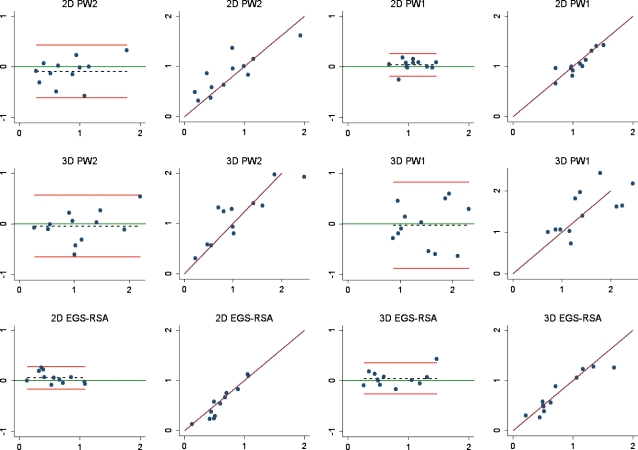
Bland-Altman plots and scatter plots, with lines of equality for repeatability measures for each of the 3 methods. Bland-Altman plots (columns 1 and 3); x-axis: average of two measurements; y-axis: the difference between 2 measurements (y = measurement 1 – measurement 2); red lines: 95% limits of agreement; dashed line: bias from 0; solid green line: y = 0 line; dots: individual double measures. Scatter plots (columns 2 and 4); x-axis: first measurement; y-axis: second measurement; maroon lines: lines of equality; EGS-RSA: radiostereometric analysis using sphere models; PW1: PolyWare using only the final follow-up radiographs; PW2: PolyWare using the postoperative and final follow-up radiographs.

Repeatability evaluated within methods revealed no clinically relevant or statistically significant bias between any 2 pairs of radiographic double examinations of PE wear. The 2D intra-method repeatability (LOA, mm) was ± 0.22, ± 0.23, and ± 0.53 for PW1, EGS-RSA, and PW2, respectively. The 3D intra-method repeatability (LOA, mm) was ± 0.31, ± 0.62, and ± 0.87 for EGS-RSA, PW2, and PW1, respectively (Table 2 and Figure 3). The relative repeatability between 2D PW1 and 2D EGS-RSA (the “gold standard”) was 1.02 (p = 0.95) (Table 3). Precision (95% CI, mm) was 0.14, 0.26, and 0.29 for the x-, the y-, and the z-axis, respectively.

**Table 2.** Repeatability of radiographic double-examination wear measurements for the 3 methods

Analysis method	Mean (range) (mm)	SD_dif-intr **a**_	Bias **^b^** (±LOA) **^c^**(mm)	95% CI **^d^**(mm)	p-value **^e^**
*2D measurements*					
PW2 **^f^**	0.80 (0.28–1.78)	0.26	-0.09 (±0.53)	-0.26–0.08	0.25
PW1 **^g^**	1.07 (0.69–1.47)	0.11	0.04 (±0.22)	-0.04–0.11	0.29
EGS-RSA **^h^**	0.60 (0.13–1.09)	0.11	0.06 (±0.23)	-0.02–0.13	0.11
*3D measurements*					
PW2	1.12 (0.27–2.20)	0.31	-0.05 (±0.62)	-0.25–0.15	0.61
PW1	1.48 (0.86–2.31)	0.44	-0.03 (±0.87)	-0.31–0.25	0.82
EGS-RSA	0.75 (0.26–1.47)	0.16	0.05 (±0.31)	-0.05–0.15	0.33

**^a^** SD_dif-intra_ is the random variation within a method comparing double examinations.

**^b^** Bias: systematic variation within a method.

**^c^** LOA: limits of agreement around the bias (95% prediction interval = SD_dif-intra_ x 1.96).

**^d^** 95% confidence interval for the bias.

**^e^** p value (paired t-test) bias between methods.

**^f^** PW2: PolyWare PE wear analysis using the postoperative and final follow-up radiographs.

**^g^** PW1: PolyWare PE wear analysis using only the final radiographic follow-up radiographs.

**^h^** EGS-RSA: radiostereometric analysis of PE wear using sphere models (the “gold standard”).

**Table 3.** Comparison of repeatability and concurrent validity between methods

Analysis method	Repeatability		Concurrent validity			
	Repeatability ratio**^a^**	p-value**^b^**	SD_dif-intr_**^c^** (mm)	Bias**^c^** (±LOA)**^e^** (mm)	CI 95% of true bias **^f^** (mm)	p-value**^g^**
*2D measurements*						
PW1 **^h^** relative to EGS-RSA **^i^**	^i^1.02	0.95	0.27	0.48 (±0.55)	0.30–0.65	< 0.001
PW2 **^j^** relative to. EGS-RSA	2.32	< 0.001	0.44	0.21 (±0.89)	-0.08–0.49	0.14
PW1 relative to PW2	2.36	< 0.01	0.34	0.27 (±0.68)	0.05–0.49	0.02
*3D measurements*						
PW1 relative to EGS-RSA	2.80	0.002	0.56	0.73 (±1.13)	0.37–1.09	< 0.001
PW2 relative t. EGS-RSA	2.00	0.03	0.53	0.36 (±1.06)	0.03–0.70	0.04
PW1 relative to PW2	1.40	0.28	0.45	0.36 (±0.90)	0.08–0.65	0.02

**^a^** Repeatability ratio: ratios of variance.

**^b^** p-value: test of variance between methods (F-test).

**^c^** SD_dif-inter_: random variation from the 2 different methods.

**^d^** Bias: systematic variation between methods.

**^e^** LOA: limits of agreement around the bias (95% prediction interval = SD_dif-inter_ x 1.96).

**^f^** 95% confidence interval for the bias.

**^g^** p-value (paired t-test) bias between methods.

**^h^** PW1: PolyWare PE wear analysis using only the final follow-up radiographs.

**^i^** EGS-RSA: radiostereometric analysis of PE wear using sphere models (the “gold standard”).

**^j^** PW2: PolyWare PE wear analysis using the postoperative and final follow-up radiographs.

**Figure 4.**
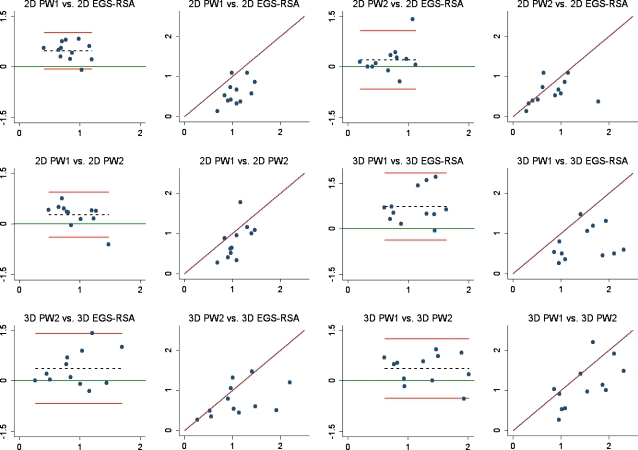
Bland-Altman plots and scatter plots with lines of equality for concurrent validity between the 3 methods. Bland-Altman plots (columns 1 and 3); x-axis: average of the measurements of 2 methods; y-axis: difference between measurements of 2 methods; red lines: 95% limits of agreement; dashed line: bias from 0; solid green line: y = 0 line; dots: individual double measures. Scatter plots (columns 2 and 4); maroon lines: lines of equality; EGS-RSA: radiostereometric analysis using sphere models; PW1: PolyWare using only the final follow-up radiographs; PW2: PolyWare using the postoperative and final follow-up radiographs.

Concurrent validity showed a statically significant (p < 0.04) bias between all pairwise comparisons of methods, except between 2D PW2 and 2D EGS-RSA. The 2D inter-method concurrent validity (LOA, mm) was ± 0.55, ± 0.89, and ± 0.68 for PW1 relative to EGS-RSA, PW2 relative to EGS-RSA, and PW1 relative to PW2, respectively. The 3D inter-method concurrent validity (LOA, mm) was ± 1.13, ± 1.06, and ± 0.90 for PW1 relative to EGS-RSA, PW2 relative to EGS-RSA, and PW1 relative to PW2, respectively (Table 3 and Figure 4).

The total investment costs were €132,982 for RSA, €7,217 for PW2, and €2,052 for PW1 using only the final radiographic follow-up (Table 4). The total staff costs for the 12 patients in this study were €1,644 for RSA, €1,068 for PW2, and €612 for PW1 (Table 4).

**Table 4.** Marginal cost analysis in € (euros) for polyethylene wear analysis by EGS-RSA and with PolyWare as used in this study. For easy comparison, the staff costs are listed per patient

Cost area	EGS-RSA **^a^**	PW2 **^b^**	PW1 **^c^**
*Investment costs (1 time)*			
Additional X-ray equipment	80,604	0	0
Calibration box	10,075	0	0
A3 transparency scanner	0	5,165	0
Software	34,595	510	510
Education of radiographer	1,542	0	0
Education of research assistant	6,166	1,542	1,542
Total investment costs	132,982	7,217	2,052
*Staff costs per patien*t			
Radiographer	35	12	12
Research assistant	102	77	39
Total staff costs per patient	137	89	51

^a^ EGS-RSA: radiostereometric analysis of PE wear using sphere models (2 stereo radiographs were used).

^b^ PW2: PolyWare PE wear analysis using the postoperative and final follow-up plain radiographs.

^c^ PW1: PolyWare PE wear analysis using only the final radiographic follow-up plain radiographs.

## Discussion

Although RSA is considered to be the most accurate and precise analysis method for PE wear (the gold standard) (Ilchmann et al. 1995, von Schewelov et al. 2004), many radiographic in vivo studies, especially retrospective studies, have been restricted to wear measurements on plain radiographs. Several computer-assisted methods for assessment of PE wear on plain radiographs are available (von Schewelov et al. 2004, McCalden et al. 2005, Geerdink et al. 2008), but few have been compared clinically with RSA (Ilchmann et al. 1992, 1995, von Schewelov et al. 2004, Bragdon et al. 2006), and to our knowledge no previous studies have evaluated the concurrent validity of RSA and the commonly used PolyWare method for plain radiographs (Devane and Horne 1999). Specifically, we wanted to determine whether it was more accurate (in agreement with RSA) to use only the final radiographic follow-up or to use both the postoperative and the final radiograph follow-ups with the PolyWare method (Stilling et al. 2009b).

Several variables in the clinical set-up may, in theory, influence the amount of wear measured. Small changes in the radiographic set-up from follow-up to follow-up, under- or overexposure of radiographs that can affect the quality and sharpness of the component borders, patient position and leg rotation, body size and soft tissue mass of the patients, and angulations and size of components are just some of the variables that may affect clinical radiographs. Wear measurements based on uncalibrated plain radiographs would naturally be more sensitive to these changes than calibrated stereo radiographs. Despite all these potential problems with plain radiographs, we did not exclude any patients or radiographs because the border of the femoral head was sufficiently visible in all radiographs.

When only the final follow-up plain radiograph (PW1) is used to estimate wear, the primary position of the femoral head in each patient (zero wear) is assumed by the PolyWare software based on CAD-based knowledge of the cup and head, and the keyed-in information on sizes. For PW2 and RSA, the postoperative radiographs provide the baseline. The algorithms for determination of wear by use of plain or stereo radiographs are not identical. Consequently, exact agreement between PE wear measurements based on different angle radiographs evaluated with different software packages cannot be anticipated, but some similarity can be expected. Both EGS-RSA and PolyWare are shadow-casting methods (Collier et al. 2003), and PolyWare relies on the marking of a beam center in the radiographs. We used only pelvic anteroposterior plain radiographs at postoperative and final follow-up; thus, the center of the ray should have been similar at different follow-ups. The resolution of the scanned primary hard copy plain radiographs and the follow-up digital radiographs we used followed the recommendations in the instruction manuals.

The radiographic set-up and the leg of the patient were repositioned between the double-examination radiographs in our study, and the calculated inter-method repeatability therefore reflects the contribution of variance from the radiographic set-up, the leg position, and the method of PE wear analysis. All 3 methods had small biases (range -0.09–0.06 mm), which were of no clinical or statistical significance. The best intra-method repeatability was obtained with 2D PW1 and 2D EGS-RSA, with approximate limits of agreement of ± 0.22 mm and ± 0.23 mm. Repeatability for all the 2D PE methods of wear measurement had limits of agreement below ± 0.5 mm, whereas repeatability of all 3D PE methods of wear measurement had limits of agreements above ± 0.5 mm.

In a clinical study, Digas et al. (2003) assessed double examinations of 45 patients and reported that precision absolute mean ± 2.7 SD (99% CI) for the 3D total was 0.22 mm. This is somewhat better than our observation for 3D EGS-RSA (LOA: ± 0.31 mm). These authors also reported translational precision of marker-based RSA to be 0.13 mm for the transverse axis, 0.10 mm for the longitudinal axis, and 0.22 mm for the sagittal axis. Röhrl et al. (2004) evaluated double examinations of patients with slight repositioning between exposures and found a longitudinal axis precision of 0.15 mm (95% CI). We used a model-based RSA method and observed a similar precision (95% CI) for the x-axis (0.14 mm) but a poorer precision for the y- and z-axis (Kaptein et al. 2006). It has already been emphasized that a 3D precision is mathematically difficult to present, as the precisions of the different directions cannot easily be added (Ryd 1986). Yet, this was necessary for a direct comparison of the repeatability of RSA and PolyWare.

In a retrieval study, PolyWare has been shown to underestimate 2D linear wear by 20% and dimensional 3D wear by 18% (Hui et al. 2003). We found the opposite tendency; that is, overestimation of wear by PolyWare in comparison to EGS-RSA as the gold standard. The relative mean difference between the 2D and 3D PE wear measured by PolyWare using two radiographic follow-ups (PW2) and EGS-RSA was 21% and 30%, respectively. Comparing PolyWare using one radiographic follow-up (PW1) and EGS-RSA, the relative difference for 2D and 3D wear was even larger (40% and 46%, respectively). As a consequence of these large differences in measured mean wear, we only established concurrent validity of the mean bias with EGS-RSA and PW2 based on statistical testing. However, the systematic variation (bias) can be corrected for when known, whereas the random variation cannot, and thus the methods with the concurrent smallest LOA are the ones in closest agreement. In our study, this was EGS-RSA and PW1.

A clinical threshold of interest for the detection of PE wear that leads to long-term osteolysis and implant failure has been established to be 0.2 mm/year (Dowd et al. 2000, Sochart 2001). This is at the lower limit of clinically measureable wear with the best 2D wear methods used in our study. When total wear measurements close to 0.2 mm are of interest (i.e. cross-linked liners at medium-term follow-up), the images should be analyzed several times, with the average value representing the true value (Vickers 2003). For PE wear analysis, however, the number of repeat wear measurements that is optimal is not known at present. Using the most accurate method (EGS-RSA), the medium-term wear rate was 0.12 mm/year, which is in accordance with a recent report (Skoldenberg et al. 2009). We have previously determined the medium-term PE wear rate (0.25 mm/year) in similar ArCom PE liners articulated with 28-mm cobalt-chromium femoral heads by wear analysis on serial radiographs (Stilling et al. 2009a). Later, we were able to show that the use of serial radiographs for wear analysis with PolyWare results in an increased amount of measured wear (Stilling et al. 2009b), which explains the higher wear rate we found in ArCom PE.

Assessing concurrent validity, the mean PE wear measured with PolyWare (PW1 and PW2) was greater than wear measured by EGS-RSA. This is similar to the report of Bragdon et al. (2006) who compared marker-based digital RSA and the Martell method on plain radiographs. They suggested a calculation and comparison of the steady-state wear between methods. In our patient series no 1- or 2-year radiographic follow-ups were available, so this was not possible.

The accuracy of 2D PE wear measurement by the EGS-RSA method was recently shown to be in very good agreement with the true wear (Stilling et al. 2009b). Thus, based on the present results, the use of only the final plain radiographic follow-up with the PolyWare method (PW1) comes within ± 0.55 mm of the true value. This is sufficient for comparative studies assessing differences between 2 groups, and if desired, the systematic error can be corrected for. Furthermore, limiting the assessed plain radiographs to the final follow-up will improve repeatability and also provide the chance of good-quality digital radiographs. Also, it permits definition of a pre-study protocol for the last follow-up radiographs, thus ensuring that there is less projection variation between radiographs in a retrospective clinical study targeting PE wear. This could also reduce the number of patients needed for evaluation.

The marginal cost analysis favors PolyWare over RSA concerning both investment costs and staff costs; however, some adjustments of the costs shown may be needed in another institution depending on the additional equipment needed. PE wear analysis with PolyWare, where only the final (and digital) radiograph is used, is the lowest priced method overall. However, because PolyWare is a less precise method than RSA, a 2–3 times larger sample size will be needed for this method (Stilling 2009), which evens out the staff costs for a prospective clinical study with the 2 methods. Yet, investment costs are 20 to 60 times more expensive for RSA, and to be cost-effective the RSA system should be used for more than 1 study. Furthermore, and something that was not included in the marginal analysis, plain radiographs are needed for documentation after surgery, whereas stereo radiographs are additional and therefore add to the total radiation dose per patient studied.

We expect our findings to have good external validity and to be applicable to good-quality radiographs of various brands of hemispheric metal shells with polyethylene liners and metal femoral heads. The PolyWare method using only final radiographic anteroposterior images is inexpensive and easy to use, is applicable for 2D wear measurements above 0.5 mm in total, and offers a simple and fast set-up that is applicable for the assessment of PE wear in most hospitals. The PolyWare method using only final radiographic anteroposterior images has a clinical repeatability similar to that of EGS-RSA (“the gold standard”) and is ideal for retrospective research because it alleviates the need for baseline images that are often lost, stored in hard copy, and of variable quality. For assessment of low PE wear (i.e. with new cross-linked liners), PolyWare software does not supply the accuracy required, and for such situations we recommend RSA. For assessment of medium-term or long-term wear measurements in larger groups of patients, the PolyWare method is optimal, simple, and in relatively close agreement with the gold standard of RSA.
